# Conformational Selection Mechanism Provides Structural Insights into the Optimization of APC-Asef Inhibitors

**DOI:** 10.3390/molecules26040962

**Published:** 2021-02-11

**Authors:** Xinheng He, Ning Huang, Yuran Qiu, Jian Zhang, Yaqin Liu, Xiao-Lan Yin, Shaoyong Lu

**Affiliations:** 1 Key Laboratory of Cell Differentiation and Apoptosis of Chinese Ministry of Education, Department of Pathophysiology, School of Medicine, Shanghai Jiao Tong University, Shanghai 200025, China; hexinheng@sjtu.edu.cn (X.H.); qiuyuran.georgina@sjtu.edu.cn (Y.Q.); methane02@163.com (J.Z.); 2 Zhiyuan Innovative Research Center, Shanghai Jiao Tong University, Shanghai 200240, China; 3 Northern Huashan Hospital, Fudan University, Shanghai 201907, China; huangning750024@163.com; 4 Medicinal Chemistry and Bioinformatics Center, School of Medicine, Shanghai Jiao Tong University, Shanghai 200025, China; 5 Department of Radiotherapy, Changhai Hospital (Hongkou District) Affiliated to Naval Medical University, Shanghai 200081, China

**Keywords:** APC-Asef, protein-protein interactions (PPIs), molecular dynamics (MD) simulations, protein dynamics, peptide drug design

## Abstract

Metastasis is the major cause of death in colorectal cancer and it has been proven that inhibiting an interaction between adenomatous polyposis coli (APC) and Rho guanine nucleotide exchange factor 4 (Asef) efficaciously restrain metastasis. However, current inhibitors cannot achieve a satisfying effect in vivo and need to be optimized. In the present study, we applied molecular dynamics (MD) simulations and extensive analyses to apo and holo APC systems in order to reveal the inhibitor mechanism in detail and provide insights into optimization. MD simulations suggested that apo APC takes on a broad array of conformations and inhibitors stabilize conformation selectively. Representative structures in trajectories show specific APC-ligand interactions, explaining the different binding process. The stability and dynamic properties of systems elucidate the inherent factors of the conformation selection mechanism. Binding free energy analysis quantitatively confirms key interface residues and guide optimization. This study elucidates the conformation selection mechanism in APC-Asef inhibition and provides insights into peptide-based drug design.

## 1. Introduction

As the primary cause of death from cancer, metastasis has been regarded as a promising therapeutic target of carcinoma for a long time since controlling it remains challenging clinically [1,2]. Protein-protein interactions (PPIs) modulates a good deal of a biological process including cell invasion and adaption [3]. Thus, the inhibition of PPIs serving a key role in metastasis has been considered an anti-cancer strategy of interest [4,5,6]. In particular, the truncated mutant adenomatous polyposis coli (APC) protein interacts with Rho guanine nucleotide exchange factor (GEF) 4, termed Asef. The interaction disrupts the intramolecular inhibitive regulation of Asef GEF activity, which promotes the colorectal cancer cell migration by stimulating the small Rho-like GTPase signaling [7,8,9]. The interfering of APC or Asef has also been proven to inhibit colorectal cancer cell migration [10]. Hence, abrogating the APC-Asef interaction paves the way for anti-cancer drug design [9,11].

**Figure 1:**
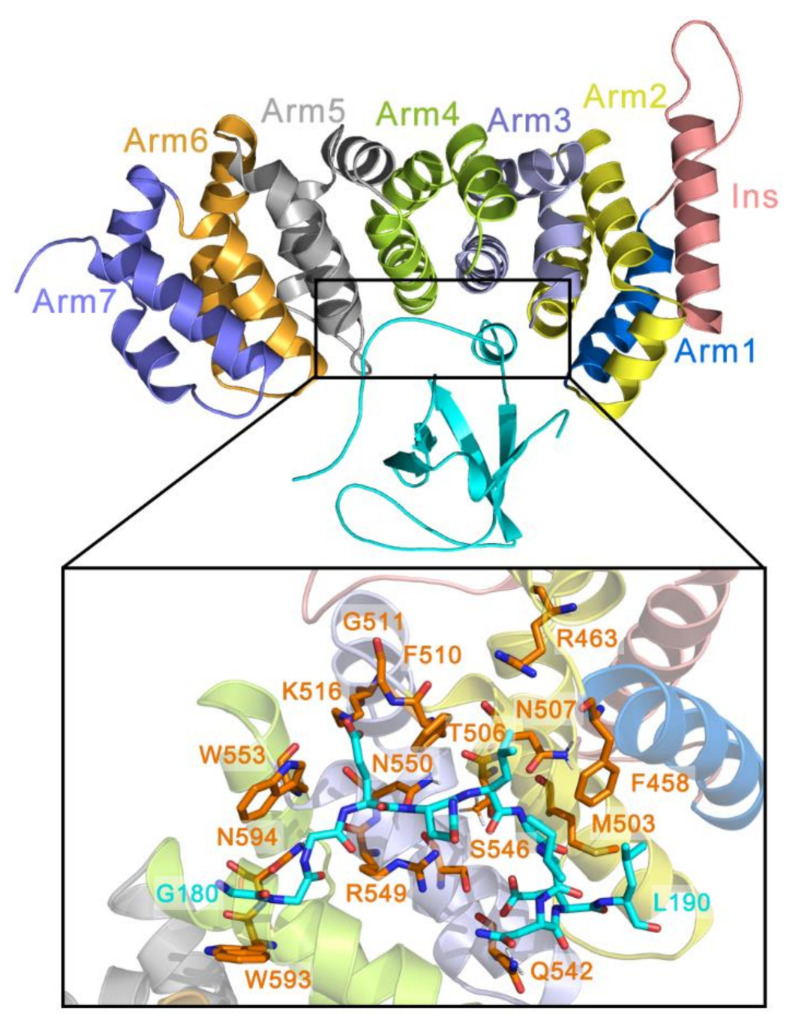
The main composition of the APC-Asef complex with a close-up view of APC-Asef interaction. In both the top and bottom subplot, Ins (salmon), Arm1 (blue), Arm2 (yellow), Arm3 (light purple), Arm4 (green), Arm5 (gray), Arm6 (orange), and Arm7 (purple) are depicted as cartoons. Asef residues on the binding interface and APC pocket residues are shown in cyan and orange sticks, respectively.

The full-length human APC protein contains 2843 amino acids and multiple secondary structures, including an oligomerization domain (OD), an armadillo repeat (ARM) domain, the repeat region for β-catenin and Axin binding, and the basic domain. Truncated APC protein is only composed of the region preceding ARM (hereafter, referred to as PreARM) and ARM, which are both highly conserved [8]. PreARM is composed of Arm1, Arm0 (both are not included in our systems), and an insertion helix (Ins), while ARM contains Arm1–7 whose basic structures are helixes and loops, as shown in Figure 1. The Asef protein consists of the APC binding region (ABR) for ARM binding, Src homology 3 domain (SH3), Dbl homology domain (DH), and pleckstrin homology domain (PH) [7,8]. The interface of APC and Asef is mainly constituted by APC Arm2–5 and Asef 180–190 (Figure 1).

It has been reported that peptide inhibitors, such as MAI-108 in Reference [9], disrupt the interaction between ARM and ABR and effectively block the formation of the APC-Asef complex, which provide insights into the development of novel therapeutics toward colorectal cancer. However, current inhibitors have an IC_50_ of around 200 nm and it is difficult for some to cross the membrane and reach their target [9,11,12]. Meanwhile, static crystallographic structures limited our view of the dynamic nature of the APC-inhibitor interactions [13,14]. It is, therefore, imperative to study the conformational ensemble of APC, APC-Asef, and APC-inhibitor systems in order to optimize current modulators from a dynamic perspective.

As a powerful approach for achieving this vision, molecular dynamics (MD) simulations have been employed for many proteins related to cancer and provides insights into their conformational ensemble [15,16,17]. With the help of MD simulation, we can observe the conformational space of APC and discover important residues in the interaction between APC and inhibitors from a dynamic viewpoint. Drawn from MD simulations, protein dynamics are also crucial in inhibitor improvement, such as increased binding affinity and effect [18]. Hence, we conducted MD simulations for apo and holo APC systems to provide insights into inhibitor optimization. The results reveal two distinct conformations exist spontaneously in the APC ensemble, which is stabilized by MAI-108 and Asef, respectively. The conformation selection mechanism is further explored by the structural analyses in detail. Meanwhile, combining analysis of the stability and binding free energy, we confirmed the results based on structure and identified several key interaction residues such as R463, R549, and W593, providing targeted improvement suggestions. Taken together, the present study shed a light on the binding mechanism of APC-Asef inhibitor and showed guidelines for structure-based inhibitor optimization.

## 2. Results

### 2.1. APC Covers a Wide Swath of Conformation Space Without a Ligand

**Figure 2:**
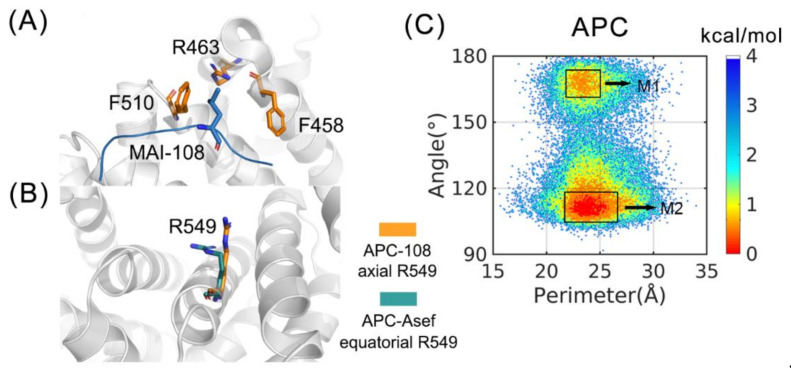
Free energy landscape of APC and corresponding parameters. (**A**) The hydrophobic pocket in the APC-108 structure. F510, R463, F458, and L185 are shown in sticks. (**B**) Different R549 in APC-108 and APC-Asef crystal structures. (**C**) The free energy landscape of APC. Different major conformations are labeled as M1 and M2. x coordinates are the perimeter of the triangle formed by the Cα of the hydrophobic pocket F510, R463, and F458, while y coordinates are the angle formed by the guanidino carbon, Cγ, and Cα of R549. See part 4.3 for details.

In order to elucidate the protein dynamics of APC-ligand complexes and optimize the peptide inhibitor of APC-Asef interaction, 200 ns conventional MD simulations were employed on APC, APC with MAI-108 (henceforth APC-108), APC-Asef, and APC with the heptapeptide on the Asef interface (henceforth, APC-ΔAsef). After simulations, we compared the crystal structures of APC-Asef and APC-MAI-108 to find rational parameters to measure the conformation ensembles. Composed of F510, R463, and F458, the hydrophobic pocket interacts with L185 and contributes to the high binding affinity of an inhibitor (Figure 2A) [9,11]. Additionally, as shown in Figure 2B, R549 changes its conformation from an equatorial state in APC-Asef to an axial state in APC-MAI-108. The axial conformation also improves the effect of the inhibitors as a critical element [8,9]. Thus, we calculated the size of the hydrophobic pocket and the bend degree of R549 in the APC system in order to measure the conformational space for ligand binding (Figure 2C).

Depicted by Figure 2C, APC has a broadly distributed population, which is classified into two dominant energy basins. M1 includes 12.64% snapshots and locates at around (23.5 Å, 170°), while M2 consists of 37.52% points and situates at about (24.5 Å, 110°). Clearly, the two major conformations are separated by the bend degree of R549. It suggests that apo APC naturally has equatorial and axial R549 and they can transform into each other. In addition, M1 and M2 represent a stable hydrophobic pocket with a perimeter of 24 Å. However, the remaining 49.84% trajectory is sporadically distributed at points out of M1 and M2, mainly on the left and right sides of the energy basins. Thus, the hydrophobic pocket fluctuates largely and exists as an unstable conformation in half of the simulation under the apo condition. Collectively, the ensemble of APC spontaneously covers broad conformational space and has two major conformers, which are indicative of an inhibitor design strategy in which ligands selectively stabilize a specific conformer.

### 2.2. Asef and ΔAsef Restrain APC Conformation in Ligand-Incompetent M2

**Figure 3:**
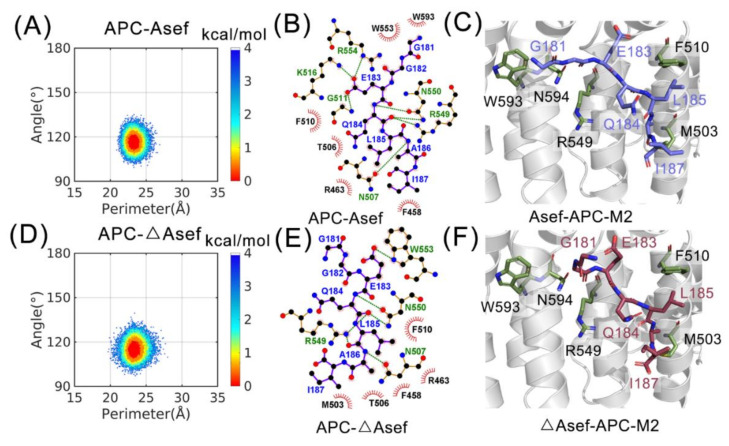
Free-energy landscapes of APC-Asef (**A**), and APC-ΔAsef (**D**) systems with corresponding interactions between APC and interface heptapeptide in (**B**,**E**). Green-dashed lines represent hydrogen bonds, while red arcs show the hydrophobic contacts. Structural superimposition of APC-Asef to APC-M2 (**C**), and APC-ΔAsef to APC-M2 (**F**). Holo APCs are neglected for clarity in (**C**,**F**).

Besides APC itself, we also measured the conformational space of APC-ligand complexes as a means to explore the effect of ligands. Using the same parameters as APC, in Figure 3A,D shows the conformation ensemble of APC-Asef and APC-ΔAsef, respectively. The energy basin of APC-Asef locates at (24 Å, 113°) while APC-ΔAsef has an energy basin at (24 Å, 110°). Of note, they both select the M2 conformer in the APC ensemble. To establish the interaction model of the APC-ligand, we clustered the representative structure of APC M2, APC-Asef, and APC-ΔAsef. Intermolecular interfaces in the two major structures are depicted in Figure 3B,E with the help of Ligplot+ [19]. Then, the holo structures are superimposed to apo APC in Figure 3C,F to unravel the conformation selection mechanism in detail.

From the perspective of the free energy landscapes, Figure 3D has a number of points scattered at the left and right of the energy basin, but Figure 3A does not have these points. Hence, horizontal fluctuation in APC-Asef is weaker than in APC-ΔAsef. In terms of R549, the bend degree in APC-ΔAsef also tends to fluctuate more in the landscape. Totally, ascribed to different APC-ligand interactions, Asef constrains conformational space in M2 stronger than ΔAsef.

The variation of ligand leads to the change of the conformational ensembles. With complete residues, Asef extends from the binding interface and residues before G181 keeps it far from E183 (Figure 3C). However, in ΔAsef, G181 contracts back and forms an intramolecular hydrogen bond with E183, leading to a decrease of the hydrophobic interactions between E183 and F510 (Figure 3F). Meanwhile, the change from a peptide bond to carboxyl in the C-terminal promotes the hydrophilic properties of the ligand, further damaging the hydrophobic pocket, especially F458 (Figure 3B,E). As for R549, it tends to interact with the middle part of the peptide in the APC-ligand complex, such as L185 and A186 (Figure 3B,E). Shown in the M2-ligand complex, however, Q184 prevents R549 from interacting with other residues (Figure 3C,F). Thus, R549 needs to overcome the steric hindrance of Q184 before an interaction, which hinders the binding of ligands. In addition, a large sidechain of Q184 inhibits the change of the R549 bend degree, contributing to the equatorial state of R549. Located far away from the ligands, equatorial R549 interacts with inhibitors weakly. Additionally, in the N-terminal of ligands, hydrophobic contacts with W553 and W593 are replaced with a hydrogen bond with W553 from APC-Asef to APC-ΔAsef (Figure 3B,E). Since hydrophobic tryptophan conflicts with polar contact, the interaction in APC-ΔAsef is weaker. In total, the composition of ligands determines the conformational ensemble of APC-ligands and detailed interactions reflect that the M2 conformer is incompetent for inhibitor binding.

### 2.3. MAI-108 Controls APC in Ligand-Competent M1 Conformation

**Figure 4:**
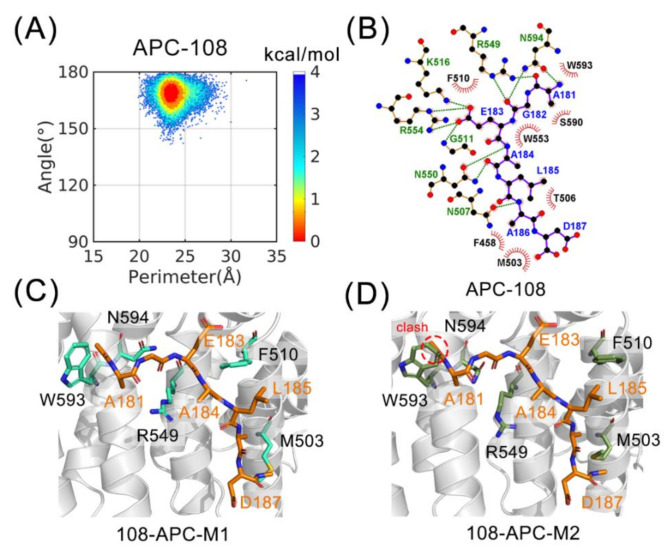
(**A**) Free-energy landscape of APC-108. (**B**) The 2D visualization of the interface of APC-108. (**C**) Structural superimposition of APC-108 to APC-M1. (**D**) Structural superimposition of APC-108 to APC-M2.

To explain the inhibiting mechanism and provide insights into the optimization of MAI-108, we also depicted the free energy landscape of APC-108 in Figure 4A. Compared with Asef and ΔAsef, MAI-108 changes 3 residues of 7 but stabilizes APC in a totally different conformation M1. For the elucidation of the binding mechanism, receptor-ligand interactions in the representative APC-108 structure are shown in Figure 4B and the alignments between APC-108 and APC-M1 (Figure 4C). APC-108 and APC-M2 (Figure 4D) are also depicted.

In Figure 4A, the energy basin of APC-108 is situated at (24 Å, 170°) with axial R549. Meanwhile, APC-108 has a broad distribution of the x coordinate in the landscape, suggesting relative flexibility of the hydrophobic pocket with MAI-108. Comprehensively, with a ligand bound, APC stops transforming between the two conformations M1 and M2, indicating that ligands selectively interact with distinct conformation and prevent APC from transformation. Therefore, the inhibitors shift the conformation ensemble of APC into a different state, which implies their mechanism in a conformation selection way.

Considering the reason why APC-108 forms such a conformation ensemble, the key points are the differences between M1 and M2 and the mutated residues from Asef to MAI-108. R549 takes an axial and equatorial state in M1 and M2, respectively, confirming that the results of the cluster represent the corresponding conformation. F510 moves toward the interface in M1 but turn around in M2, which reflects that its hydrophobic interaction is stronger in M1 than M2. M503 also extend its side chain out to increase the hydrophobic contact area in M1, rather than a folded status in M2. Intriguingly, W593 and N594 slightly move away from the interface in M1 in order to keep a rational distance for interaction but not clash (Figure 4C,D). Hence, M1 is more suitable for accepting a ligand, and MAI-108 selects and stabilizes M1. In the optimization process, ligand-M1 interaction can be potentiated by stabilizing axial R549 or pulling W593 outward. In addition, disrupting M2 conformation, such as extending M503 and twisting F510, contributes to the binding of inhibitors.

In the field of residue composition, the major change is caused by Q184A. The interactions between Q184 and R549 also push Q184 away and tend to confine R549 in an equatorial state (Figure 3C,F). However, in MAI-108, A184 is small enough for a space acceptable to R549 (Figure 4C). Axial R549 interacts with N-terminal residues, like A181, in APC-108 (Figure 4B), which prevents the steric hindrance to interact with middle residues. Thus, axial R549 is more suitable for inhibitor binding, suggesting that the volume of 184 can be shrunken more to stabilize axial R549 during optimization. In addition, the sidechain of A181 clashes with W593 in an M2 conformation, further reflecting that MAI-108 conflicts with M2 (Figure 4D). Collectively, the data indicate that APC-M1 and MAI-108 adapt to each other, and APC-M2 fits Asef and ΔAsef more, confirming a conformation selection mechanism in inhibitor binding.

As for the fluctuation of a hydrophobic pocket in APC-108, I187D provides an extra hydrophilic part in the ligand and further increases the polarity of the C-terminal, causing a larger fluctuation in F458 nearby (Figure 4C,D). It is inferred that, to increase the length of the sidechain, the hydrophobicity of the C-terminal may facilitate the binding of the inhibitor and its ability to control the ensemble. The inhibitor penetrability for the cell membrane can also be improved. Following this rule, Mutant D187E has been shown to promote the binding affinity of the ligand [9]. In conclusion, structural analyses reveal the mechanism of inhibitor binding and provide insights into optimization, but the dynamic properties of systems still need to be considered.

### 2.4. The Fluctuation Analysis Confirms the Conformation Selection Mechanism

**Figure 5:**
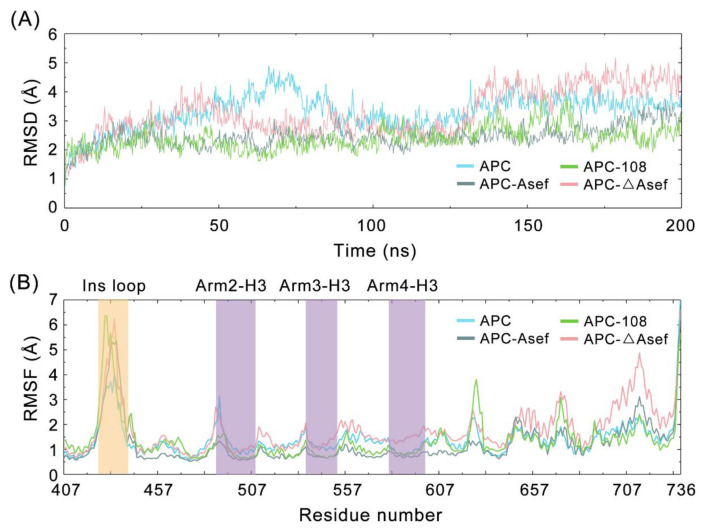
(**A**) The Cα RMSDs of APC residues along the whole MD trajectories for APC (blue), APC-108 (green), APC-Asef (gray), and APC-ΔAsef (pink), referred to the starting structure. (**B**) RMSFs of APC Cα atoms for APC (blue), APC-108 (green), APC-Asef (gray), and APC-ΔAsef (pink) systems. Yellow and purple rectangles represent a highly fluctuated domain and major ligand-binding domains, respectively.

According to the comparison among systems, the conformation selection mechanism was identified but needs to be explained further. Thus, we explored the overall and individual dynamic properties of APC residues by calculating the root mean square deviation (RMSD) and root mean square fluctuation (RMSF) value of each snapshot (Figure 5A,B). The initial increasing tendency of RMSD stops at around 25 ns, indicating that all systems reach equilibrium at around 25 ns. Thus, the following analyses were based on the last 175 ns.

On the period of 25–200 ns, the average RMSD of APC and APC-ΔAsef system were 3.45 ± 0.46 Å and 3.41 ± 0.74 Å, respectively, which are higher than APC-Asef (2.49 ± 0.33 Å) and APC-108 (2.43 ± 0.39 Å). Due to the apo state of the APC system, a larger fluctuation is normal. The addition of effective inhibitors, such as Asef and MAI-108, decreases RMSD and stabilizes the APC protein. However, the fluctuation of APC-ΔAsef, especially after 130 ns (4.16 ± 0.43 Å), reflects that ΔAsef leads to instability of APC, related to its weak inhibition effect. As shown in Figure 5B, the overall shape of system RMSF variation is generally similar, and highly fluctuating loops, such as Ins loop, display a larger RMSF. Meanwhile, APC-ΔAsef shows globally higher RMSF compared to other systems, particularly in the binding domain and the terminus of APC. Hence, rational inhibitors decrease protein flexibility in contrast to ΔAsef.

Focused on the landscape parameters, the RMSF values of corresponding residues (shown in Table 1) provide a detailed explanation for the conformation selection mechanism. The movement of R549 is not clear in strong inhibitors like MAI-108 and Asef, suggesting that the ability to control the bend degree of R549 is critical in inhibitor design. From the viewpoint of the hydrophobic pocket, ΔAsef loses the interaction between E183 and F510 due to the contraction. Therefore, the fluctuation of F510 is larger than other systems. Asef globally decreases the flexibility of the pocket, corresponding to its concentrated free energy landscape. The major movement of APC-108 comes from F458 and R463, which are close to the mutation I187D, reflecting the influence of residue polarity in the C-terminal and the necessity to modify 187 to increase the binding affinity. In total, RMSFs for individual residues confirm the conclusion obtained from structural analyses.

### 2.5. Dynamic Properties Provide Detailed Information for APC-Ligand Interactions

**Figure 6:**
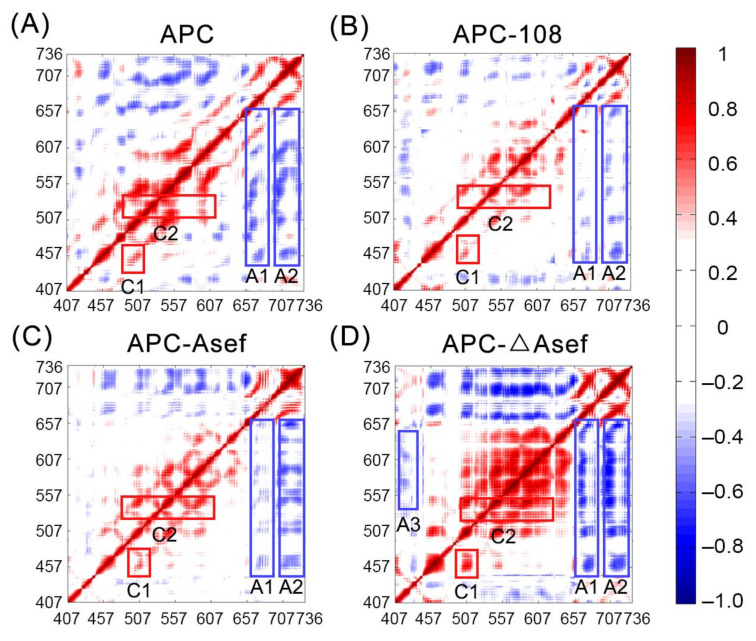
The dynamic cross-correlation matrixes (DCCM) of APC (**A**), APC-108 (**B**), APC-Asef (**C**), and APC-ΔAsef (**D**). Red rectangles (C1–C2) show important correlated areas, while blue rectangles (A1–A3) show areas with clear anti-correlations. The interactions with an absolute correlation coefficient of less than 0.3 are colored white for clarity.

Interactions between residues are fundamental for protein stability and the binding mechanism. Hence, to further explore the dynamic variation of APC with different ligands, residue interactions were analyzed by dynamic cross-correlation matrixes (DCCM) in Figure 6. DCCM is composed of correlation coefficients based on related motions between each Cα in the whole trajectory and reflects the relationship of different domains. The stronger relationship indicates a higher tendency to interact with each other. Interactions between domains are significant in the APC-ΔAsef system, as shown by the highest absolute coefficients in Figure 6D. Removing ΔAsef engenders a decrease of correlated motions, while binding of Asef further inhibits interactions between residues. As noted in Figure 6B, MAI-108 controls global interactions and mostly stabilizes the APC protein.

In each state, C1 represents the correlation movement in the area of a hydrophobic pocket composed of F510, F458, and R463. Both Asef and MAI-108 effectively dampen C1 correlation, but ΔAsef promotes it instead (Figure 6B–D). It is inferred that ΔAsef has no ability to dominate the dynamics of the interface. Rather, it strengthens the interaction between residues and increases system flexibility. On the other hand, C2 shows the interaction of R549 to the interface residues. MAI-108 weakens the C2 relationship best, which is ascribed to the hydrogen bond between axial R549, A181, and G182 (Figure 4B and Figure 6B). In Figure 6C,D, equatorial R549 shows a clear relationship with other APC residues and tends to interact with them but not the ligand. In particular, the correlation in APC-ΔAsef in this domain is even larger than the apo state (Figure 6A). It suggests that Asef and ΔAsef do not interact with equatorial R549 well, implying that the axial R549 contributes to ligand binding affinity.

The anti-correlations are mainly distributed in Arm6–7 with other areas. A1 and A2 represent the anti-correlation between Arm1–6 and Arm6 as well as Arm1–6 and Arm7, respectively. Figure 6B shows that MAI-108 quenches both A1 and A2 significantly, suggesting that MAI-108 inhibits the interaction between Arm6–7 and Arm1–6. The interactions out of the interface in APC-108 may indicate communications between the inhibitor site and other domains. Compared with MAI-108, Asef only suppresses A1 but leaves A2 similar to the apo APC system (Figure 6C). As the crystal structure of APC-Asef shows, the interface of the protein-protein interaction spreads from Arm2 to Arm6 [8]. Thus, out of the direct touching part, Asef loses its influence on the stability of APC. Without the ligand, A1 of APC shows anti-correlations on par with A2 of APC and APC-Asef (Figure 6A,C), indicative of a normal fluctuation in the apo state. Nevertheless, ΔAsef increases the anti-correlation of Arm6 and Arm7 with other domains at the same time, which substantiates that ΔAsef induces an extra relationship between domains and facilitates related movements. In addition, the emergence of A3, expressing the interaction between Ins loop and Arm3–5, further confirms the additional flexibility of APC-ΔAsef (Figure 6D). To sum up, MAI-108 and Asef quench the relationship between domains, but ΔAsef promotes the related movement instead, which is indicative of the importance to dominate interface interactions during optimization.

### 2.6. Binding Free Energy Analysis Provides Guidelines for Inhibitor Optimization

In order to guide the optimization process, besides the previously mentioned qualitative research on APC-ligand interaction, Molecular Mechanics/Generalized Born Surface Area (MM/GBSA) methods were also employed to quantitatively analyze the interaction. As shown in Table 2, the binding free energies for APC-108, APC-ΔAsef, and APC-Asef are −66.95 ± 6.94 kcal/mol, −35.18 ± 7.52 kcal/mol, and −95.52 ± 12.00 kcal/mol, respectively, which are consistent with the experimental results [9]. Specifically, the lower ΔE_vdw_ and ΔE_ele_ in APC-108 prove tighter electrostatic and van der Waals interactions of MAI-108 than ΔAsef, as shown in Figure 3E and Figure 4B. Of note, MAI-108 does not greatly increase polar solvation free energy (ΔG_P_), which keeps the advantage of ΔE_vdw_ and ΔE_ele_. Meanwhile, ΔAsef has much fewer electrostatic and van der Waals interactions with APC than Asef, indicating that the remaining part of Asef also considerably contributes to the binding. However, caused by its volume, large polar solvation free energy (ΔG_P_) in APC-Asef prevents its ΔG_binding_ from decreasing further. Thus, the optimization process should keep electrostatic and van der Waals interaction strength with APC while controlling the volume of the ligand.

**Figure 7:**
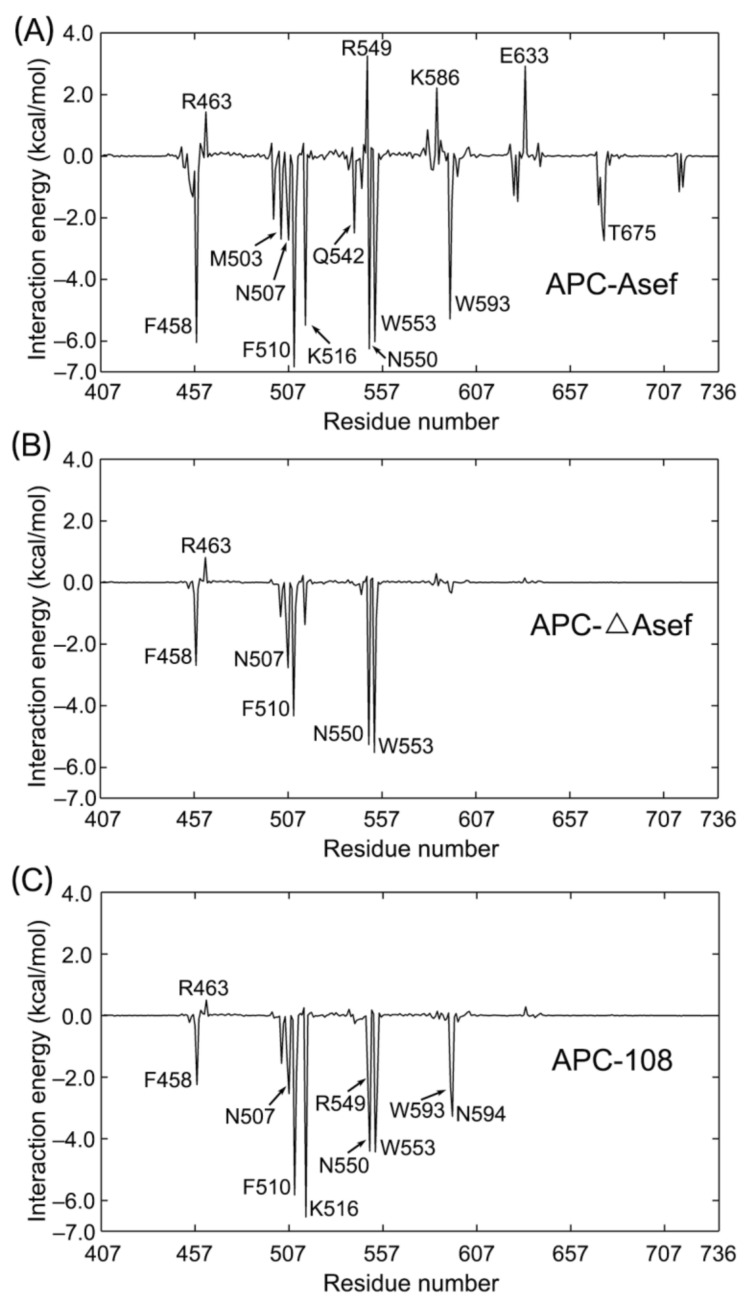
Free energy contributions of APC residues to the binding of Asef (**A**), ΔAsef (**B**), and MAI-108 (**C**). Residues with a large absolute energy value or highly different among systems are identified.

Besides the overall binding free energy analysis, we also employed energy decomposition for each system. During the decomposition, the contribution of every APC residue to the binding of ligands is quantified and plotted in Figure 7. Some critical residues show common minus free energy among the three systems. For example, N550 forms a common hydrogen bond with ligands in systems, and F458, F510, and W553 have hydrophobic contact with the ligands. These residues all decrease the binding free energy in the three systems (Figure 7). Thus, the result of MM/GBSA corresponds with our analysis based on the representative structures.

In contrast, the following residues show distinct free energy among systems. Firstly, R549 hinders APC-Asef interactions clearly (+3.25 kcal/mol, in Figure 7A), but has an insignificant effect in APC-ΔAsef (+0.20 kcal/mol, in Figure 7B), confirming that equatorial R549 serves as a negative factor for inhibitor binding. Nevertheless, changed to the axial state in APC-108, R549 brings a free energy decrease of 2.10 kcal/mol, which is indicative of its contribution for the binding effect (Figure 7C). Thus, keeping R549 axial is a must for compound design in the future. Next, as a part of the hydrophobic pocket, the energy of R463 maintains different positive values in all systems due to its polar sidechain. It is inferred that editing L185 to form a hydrogen bond with R463 but keep hydrophobic interaction with F510 and F458 may deal with the problem of R463 and increase the binding affinity. In addition, W593 and N594 interact with Asef rather than ΔAsef because ΔAsef contracts its N-terminal (Figure 7A,B). However, MAI-108 re-induce the negative binding free energy of W593 and N594, reflecting its extended conformation in the N-terminal and the match between MAI-108 and M1 (Figure 7C). By the way, out of the interface, the APC-Asef complex still has energy fluctuation, such as E633 and T675, implying that Asef also interacts with Arm5 H3 and Arm6 H3 (Figure 7A). In conclusion, energy decomposition shows important interaction residues and provides guidelines for inhibitor optimization, such as keeping R549 axial, forming hydrogen bonds with R463, and controlling the extended ligand conformation.

## 3. Discussion

The APC-Asef interaction plays a vital role in cancer invasion and metastasis. Efforts have been converged for the design of the APC-Asef inhibitor and it avoids the development of neoplasm. Here, all-atom MD simulations and extensive analyses were applied to apo, Asef bound, ΔAsef bound, and MAI-108 bound APCs in order to elucidate the mechanism of inhibition and optimize current inhibitors.

After simulations, we discovered the conformational space of apo APC and identified its two major conformers. Then, the conformation selection mechanism was unraveled by the free energy landscapes of APC-ligands. Analyses based on representative structures explain the reason why MAI-108 fits ligand-competent M1, but Asef and ΔAsef fit ligand-incompetent M2. In stability analysis, we found that APC stability is positively related to inhibitor efficiency and confirms the structural analyses. Hence, increasing the ability to maintain APC stability, especially in the interface, contributes to the optimization of inhibitors. Next, DCCM unveils the dynamic properties of systems, suggesting a different ability of ligands to control the movement of APC residues. This ability tends to provide a binding affinity. At last, binding energy analysis based on MM/GBSA quantitatively identifies important residues for binding and guide future optimization. In total, our simulations explain the mechanism of the inhibitor and provide insights for lead compound design from a perspective of protein dynamics.

Including the APC-Asef interaction, protein-protein interactions (PPIs) play a pivotal role in multiple pathological processes and serve as valuable drug targets [20,21]. However, the large pocket surface impedes the development of small-molecule inhibitors. As a result, with medium volume and structural similarity to protein, peptides are the most studied lead compounds for PPIs [22,23]. Peptide and peptidomimetic compounds have the advantages of feasible synthesis and great functional group compatibility. Nevertheless, they are also faced with some difficulties, such as low binding affinity, weak ability to cross the membrane, and proteolytic instability [9,24]. From a viewpoint of protein dynamics, the present study shows guidelines to optimize peptide inhibitors for PPIs. First, we can identify unique sidechain conformation suitable for peptide binding and strengthen the complex further. For instance, different inhibitors stabilize M1 and M2 of APC, respectively. Second, inhibitors should control the fluctuation of the protein and dominate the movement of receptor residues, thereby increasing the stability of the target protein. Third, using binding free energy analysis, residues hindering interactions can be solved in a targeted way and those promoting interactions should be kept or strengthened.

In particular, the following strategies may benefit current APC inhibitor optimization. The N-terminal mainly interacts with W593 and N594. Therefore, A181 and G182 can be replaced by a heterocycle to promote a hydrophobic interaction and attempt to form hydrogen bonds with the two residues. As for the C-terminal, changing A186 and D187 to a pure aromatic ring maintains dominant hydrophobic interactions here. These hydrophobic substituents also contribute to membrane permeability. In the middle of the ligand, the axial conformation of R549 should be further kept by polar ligand atoms, but a bulky group suppresses R549. Thus, A184S may improve the affinity of the ligand. At last, the side chain of L185 can be longer and a polar atom should be induced at its top to form a salt bridge with R463. In this way, the contact with the hydrophobic pocket can be increased and R463 may contribute to ligand binding.

Another strategy targeting PPIs is to apply the allosteric effects. Allostery represents the effect caused by modulators interacting with a site different from the functional site of biomolecules [25,26]. With a precise fine-tune ability and diverse pockets, the allosteric drug has been considered as a promising PPI modulator [27,28]. In our research, it is also worth noting that MAI-108 can influence the area without a direct interaction, but Asef loses its control of the untouched area (Arm7, A2 in Figure 6C). Thus, according to recent allosteric methodology, reversing allosteric communication may exist between the interface and the terminal of APC [29,30]. Focused on this area, the prediction of the allosteric site and the design of allosteric modulators of an APC-Asef interaction may be promoted.

## 4. Materials and Methods

### 4.1. MD Simulation Systems Setup

The crystal structures of APC-Asef (PDB ID: 3NMZ) and APC-MAI-108 (PDB ID: 5IZ8) were downloaded from the Protein Data Bank [8,9,31]. Coordinates of chain A was selected as the starting structure because of its structural integrity. By deleting the Asef in the APC-Asef system, we constructed the APC system. The APC-ΔAsef system consists of APC and residues 181–187 of Asef, whose coordinates are drawn from the APC-Asef system. Table 3 shows the system information.

### 4.2. MD Simulations

All MD simulation systems were modeled using the Amber ff03 force field due to its better performance in PPI evaluation than ff14SB [32,33]. Its deficiency of tending to form α-helix in protein folding has limited influence in our folded PPI system [34]. Each complex was solvated in a truncated octahedral transferable intermolecular potential three point (TIP3P) water box with a buffer of 10 Å around it [35]. Then, counterions Na^+^ or Cl^−^ were added to the systems for neutralization. The Particle Mesh Ewald (PME) method was invoked to calculate long-range interactions [36]. The cutoff value was set to 10 Å in all systems, as a means to treat short-range electrostatic and van der Waals forces. The size of the PME grid in each system was shown in Table 3 and the grid spacing was 1 Å. To restrain bonds containing hydrogen, the SHAKE algorithm was performed in our systems [37].

After preparations, all systems encountered two energy minimization processes. First, the coordinates of amino acids were confined with restraint of 500 kcal mol^−1^ Å^−2^, while water and counterions were minimized in 2000 steepest descent minimization cycles, and then 3000 conjugate gradient minimization cycles. Second, 4000 cycles of steepest descent minimization were applied to entire systems without any restriction, and then 6000 cycles of conjugated gradient minimization. Next, the temperatures of systems were gradually increased from 0 to 300 K in 300 ps in a canonical ensemble (NVT). After heating, NVT equilibration runs of the systems were carried out at 300 K for 700 ps. At last, 200 ns MD simulations, whose integration steps are 2.0 fs, were assigned for systems at 1 atm and 300 K. The temperatures were all controlled by Langevin Thermostat with a collision frequency of 1.0 ps^−1^ [38]. Snapshots were written out in every 5 ps. Periodic boundary conditions were performed in the four systems. CPPTRAJ suite of AMBER was applied to coordinate analysis [39]. The simulations and analyses are based on Amber16 suite [40].

### 4.3. Free Energy Landscape

A relative energy value, known as the potential mean force (PMF), was calculated to depict the free energy landscape of APC. For every snapshot, the perimeter of the triangle formed by the Cα of the hydrophobic pocket F510, R463, and F458 was defined as the x coordinate, while the angle formed by the guanidino carbon, Cγ, and Cα of R549 was the y coordinate. Then, the perimeter and angle values were set as an (x, y) pair. Using the Equation (1), the PMF values were calculated.

ΔG(x, y)  =  k_B_ × T × ln *g*(x, y) (1)

in which, k_B_ represents the Boltzmann constant (3.30 × 10^−4^ kcal/K), T is the temperature of systems (300 K), and *g*(x, y) means the normalized joint probability distribution of 200 bins of (x,y). After calculation, all energy values minus the minimum energy can obtain the relative energy value. Representative structures are extracted using the hieragglo algorithm in CPPTRAJ.

### 4.4. Dynamic Network Analysis

The inter-actions and intra-actions between APC domains were depicted by the dynamic cross correlation matrix (DCCM) [41]. DCCM is composed of correlation coefficient C_ij_, in which i and j represent each pair of Cα atoms. Calculated by Equation (2), C_ij_ reflects the correlation degree of Cα movements.

C_ij_  =  (Δr_i_ × Δr_j_)/(⟨Δr_i_^2^⟩ × ⟨Δr_j_^2^⟩)^1/2^ (2)

In the equation, Δr_i_ and Δr_j_ denote the atomic displacement vectors for Cα atoms of residues i and j, respectively. The angle brackets indicate the average calculation among entire simulations. Finally, colored matrixes in Figure 6 were used to visualize each C_ij_ and reflect the relevant movement of motifs.

### 4.5. Free Energy Calculations

MMPBSA.py plugin in AmberTools18 was applied to exploit Molecular Mechanics/Generalized Born Surface Area (MM/GBSA) in binding free energy calculations [42]. In total, the binding free energies (ΔG_binding_) of different ligands toward APC are expressed in Equation (3).

ΔG_binding_ = G_complex_ − G_apc_ − G_ligand_ (3)

Meanwhile, the second law of thermodynamics reflects that ΔG_binding_ equals to enthalpy changes (ΔH) minus the product of entropy changes and temperature (TΔS), as Equation (4) expresses.

ΔG_binding_ = ΔH − T × ΔS (4)

Usually, the system conformation entropy (−T × ∆S) was estimated by normal mode analysis with a quasi-harmonic model, but it could be omitted here because of the similarity among APC-108, APC-Asef, and APC-ΔAsef systems [43,44,45]. In addition, estimation of the conformation entropy for the protein-protein interactions remain challenging and inaccurate. Meanwhile, the difference between ΔH is large enough for the conclusion. Thus, we omitted the calculation of the −T × ∆S term and only concentrated on the relative ordering of the free energy changes.

In the simulation process, ΔH is transformed into the sum of the molecular mechanical energies (ΔE_MM_) and the solvation free energy (ΔG_solv_), according to Equation (5).

ΔH = ΔE_MM_ + ΔG_solv_ (5)

In addition, ΔE_MM_ includes the intramolecular energy (ΔE_int_, consisting of bond, angle, and dihedral energies), van der Waals energy (ΔE_vdw_), and electrostatic energy (ΔE_ele_), while ΔG_solv_ incorporates the polar (ΔG_P_) and the non-polar items (ΔG_np_). Equations (6) and (7) represent them.

ΔE_MM_ = ΔE_int_ + ΔE_vdw_ + ΔE_ele_ (6)

ΔG_solv_ = ΔG_P_ + ΔG_np_ (7)

The Generalized Born model was used to calculate ΔG_P_. ΔG_np_ was calculated based on the solvent-accessible surface area (SASA) in Equation (8).

ΔG_np_ = γSASA + b (8)

The solvation parameter γ and b were 0.00542 kcal (mol^−1^·Å^−2^) and 0.92 kcal/mol, respectively. The decomposition of the free energy into residues was subsequently carried out by the MMPBSA.py plugin [42].

## Figures and Tables

**Table 1:** Root mean square fluctuation (RMSF) values (Å) for R549, F458, R463, and F510 in APC, APC-108, APC-Asef, and APC-Asef.

Residue Number	RMSF of APC	RMSF of APC-108
R549	1.10	0.73
F458	1.31	1.08
R463	1.06	1.17
F510	1.16	0.79

**Table 2:** Free energy values (kcal/mol) for the binding of MAI-108, ΔAsef, and Asef to APC.

Energy Items	MAI-108	ΔAsef	Asef
ΔE_vdw_	−62.26 ± 3.97	−50.67 ± 3.67	−158.43 ± 9.58
ΔE_ele_	−185.94 ± 28.51	−139.41 ± 37.90	−999.05 ± 62.78
ΔG_P_	190.25 ± 24.27	161.53 ± 35.03	1083.73 ± 60.40
ΔG_np_	−9.00 ± 0.45	−6.64 ± 0.66	−21.77 ± 1.45
ΔE_MM_	−248.21 ± 28.40	−190.08 ± 38.72	−1157.48 ± 64.48
ΔG_solv_	181.25 ± 24.09	154.90 ± 34.51	1061.96 ± 59.79
ΔG_binding_	−66.95 ± 6.94	−35.18 ± 7.52	−95.52 ± 12.00

**Table 3:** Summary of the MD simulation systems.

System Name	Molecule Occupying ARM Domain	PDB ID	PME Grid Sizes (Å)
APC	None	3NMZ	108 × 108 × 108
APC-108	MAI-108 (AGEALAD)	5IZ8	108 × 108 × 108
APC-Asef	Asef	3NMZ	112 × 112 × 112
APC-ΔAsef	Residue 181–187 of Asef (GGEQLAI)	3NMZ	112 × 112 × 112

## Data Availability

The crystal structures of APC-Asef (PDB ID: 3NMZ) and APC-MAI-108 (PDB ID: 5IZ8) were downloaded from the Protein Data Bank.

## References

[1] Fidler I.J., Kripke M.L. (2015). The challenge of targeting metastasis. Cancer Metastasis Rev..

[2] Steeg P.S. (2016). Targeting metastasis. Nat. Rev. Cancer.

[3] Nero T.L., Morton C.J., Holien J.K., Wielens J., Parker M.W. (2014). Oncogenic protein interfaces: Small molecules, big challenges. Nat. Rev. Cancer.

[4] Ivanov A.A., Khuri F.R., Fu H. (2013). Targeting protein—protein interactions as an anticancer strategy. Trends Pharmacol. Sci..

[5] Schapira M., Tyers M., Torrent M., Arrowsmith C.H. (2017). WD40 repeat domain proteins: A novel target class?. Nat. Rev. Drug Discov..

[6] Li J., Xi W., Li X., Sun H., Li Y. (2019). Advances in inhibition of protein-protein interactions targeting hypoxia-inducible factor-1 for cancer therapy. Bioorg. Med. Chem..

[7] Kawasaki Y., Senda T., Ishidate T., Koyama R., Morishita T., Iwayama Y., Higuchi O., Akiyama T. (2000). Asef, A Link between the Tumor Suppressor APC and G-Protein Signaling. Science.

[8] Zhang Z., Chen L., Gao L., Lin K., Zhu L., Lu Y., Shi X., Gao Y., Zhou J., Xu P. (2012). Structural basis for the recognition of Asef by adenomatous polyposis coli. Cell Res..

[9] Jiang H., Deng R., Yang X., Shang J., Lu S., Zhao Y., Song K., Liu X., Zhang Q., Chen Y. (2017). Peptidomimetic inhibitors of APC–Asef interaction block colorectal cancer migration. Nat. Chem. Biol..

[10] Kawasaki Y., Sato R., Akiyama T. (2003). Mutated APC and Asef are involved in the migration of colorectal tumour cells. Nat. Cell Biol..

[11] Yang X., Zhong J., Zhang Q., Qian J., Song K., Ruan C., Xu J., Ding K., Zhang J. (2018). Rational Design and Structure Validation of a Novel Peptide Inhibitor of the Adenomatous-Polyposis-Coli (APC)-Rho-Guanine-Nucleotide-Exchange-Factor-4 (Asef) Interaction. J. Med. Chem..

[12] Yan X.-Q., Wang Z.-C., Qi P.-F., Li G., Zhu H.-L. (2019). Design, synthesis and biological evaluation of 2-H pyrazole derivatives containing morpholine moieties as highly potent small molecule inhibitors of APC-Asef interaction. Eur. J. Med. Chem..

[13] Narayan A.R.H., Jiménez-Osés G., Podust L.M., Montgomery J., Houk K.N., Sherman D.H., Liu P., Negretti S., Zhao W., Gilbert M.M. (2015). Enzymatic hydroxylation of an unactivated methylene C-H bond guided by molecular dynamics simulations. Nat. Chem..

[14] Amaro R.E., Baudry J., Chodera J., Demir Ö., McCammon J.A., Miao Y., Smith J.C. (2018). Ensemble docking in drug discovery. Biophys. J..

[15] Lu S., Deng R., Jiang H., Song H., Li S., Shen Q., Huang W., Nussinov R., Yu J., Zhang J. (2015). The Mechanism of ATP-Dependent Allosteric Protection of Akt Kinase Phosphorylation. Structure.

[16] Lu S., Chen Y., Wei J., Zhao M., Ni D., He X., Zhang J. (2020). Mechanism of allosteric activation of SIRT6 revealed by the action of rationally designed activators. Acta Pharm. Sin. B.

[17] Ni D., Wei J., He X., Rehman A.U., Li X., Qiu Y., Pu J., Lu S., Zhang J. (2021). Discovery of cryptic allosteric sites using reversed allosteric communication by a combined computational and experimental strategy. Chem. Sci..

[18] Latorraca N.R., Venkatakrishnan A.J., Dror R.O. (2016). GPCR Dynamics: Structures in Motion. Chem. Rev..

[19] Laskowski R.A., Swindells M.B. (2011). LigPlot+: Multiple ligand-protein interaction diagrams for drug discovery. J. Chem. Inf. Model..

[20] Rosell M., Fernández-Recio J. (2018). Hot-spot analysis for drug discovery targeting protein-protein interactions. Expert Opin. Drug Discov..

[21] Lu S., Jang H., Zhang J., Nussinov R. (2016). Inhibitors of Ras-SOS Interactions. ChemMedChem.

[22] Tsomaia N. (2015). Peptide therapeutics: Targeting the undruggable space. Eur. J. Med. Chem..

[23] Nevola L., Giralt E. (2015). Modulating protein–protein interactions: The potential of peptides. Chem. Commun..

[24] Wójcik P., Berlicki Ł. (2016). Peptide-based inhibitors of protein–protein interactions. Bioorg. Med. Chem. Lett..

[25] Lu S., Shen Q., Zhang J. (2019). Allosteric Methods and Their Applications: Facilitating the Discovery of Allosteric Drugs and the Investigation of Allosteric Mechanisms. Acc. Chem. Res..

[26] Ni D., Lu S., Zhang J. (2019). Emerging roles of allosteric modulators in the regulation of protein-protein interactions (PPIs): A new paradigm for PPI drug discovery. Med. Res. Rev..

[27] Lu S., He X., Ni D., Zhang J. (2019). Allosteric Modulator Discovery: From Serendipity to Structure-Based Design. J. Med. Chem..

[28] Lu S., Zhang J. (2019). Small Molecule Allosteric Modulators of G-Protein-Coupled Receptors: Drug–Target Interactions. J. Med. Chem..

[29] Leroux A.E., Biondi R.M. (2020). Renaissance of Allostery to Disrupt Protein Kinase Interactions. Trends Biochem. Sci..

[30] An X., Lu S., Song K., Shen Q., Huang M., Yao X., Liu H., Zhang J. (2019). Are the Apo Proteins Suitable for the Rational Discovery of Allosteric Drugs?. J. Chem. Inf. Model..

[31] Rose P.W., Bi C., Bluhm W.F., Christie C.H., Dimitropoulos D., Dutta S., Green R.K., Goodsell D.S., Prlić A., Quesada M. (2012). The RCSB Protein Data Bank: New resources for research and education. Nucleic Acids Res..

[32] Duan Y., Wu C., Chowdhury S., Lee M.C., Xiong G., Zhang W., Yang R., Cieplak P., Luo R., Lee T. (2003). A point-charge force field for molecular mechanics simulations of proteins based on condensed-phase quantum mechanical calculations. J. Comput. Chem..

[33] Wang E., Weng G., Sun H., Du H., Zhu F., Chen F., Wang Z., Hou T. (2019). Assessing the performance of the MM/PBSA and MM/GBSA methods. 10. Impacts of enhanced sampling and variable dielectric model on protein–protein Interactions. Phys. Chem. Chem. Phys..

[34] Fluitt A.M., de Pablo J.J. (2015). An Analysis of Biomolecular Force Fields for Simulations of Polyglutamine in Solution. Biophys. J..

[35] Jorgensen W.L., Chandrasekhar J., Madura J.D., Impey R.W., Klein M.L. (1983). Comparison of simple potential functions for simulating liquid water. J. Chem. Phys..

[36] Darden T., York D., Pedersen L. (1993). Particle mesh Ewald: An *N*⋅log(*N*) method for Ewald sums in large systems. J. Chem. Phys..

[37] Ryckaert J.-P., Ciccotti G., Berendsen H.J.C. (1977). Numerical integration of the cartesian equations of motion of a system with constraints: Molecular dynamics of n-alkanes. J. Comput. Phys..

[38] Adelman S.A., Doll J.D. (1976). Generalized Langevin equation approach for atom/solid-surface scattering: General formulation for classical scattering off harmonic solids. J. Chem. Phys..

[39] Roe D.R., Cheatham T.E. (2013). PTRAJ and CPPTRAJ: Software for processing and analysis of molecular dynamics trajectory data. J. Chem. Theory Comput..

[40] Case D.A., Cerutti D., Cheateham T., Darden T., Duke R., Giese T., Gohlke H., Goetz A.W., Greene D., Homeyer N. (2016). AMBER16 package.

[41] Swaminathan S., Harte W.E., Beveridge D.L. (1991). Investigation of domain structure in proteins via molecular dynamics simulation: Application to HIV-1 protease dimer. J. Am. Chem. Soc..

[42] Miller B.R., McGee T.D., Swails J.M., Homeyer N., Gohlke H., Roitberg A.E. (2012). MMPBSA.py: An efficient program for end-state free energy calculations. J. Chem. Theory Comput..

[43] Sun H., Duan L., Chen F., Liu H., Wang Z., Pan P., Zhu F., Zhang J.Z.H., Hou T. (2018). Assessing the performance of MM/PBSA and MM/GBSA methods. 7. Entropy effects on the performance of end-point binding free energy calculation approaches. Phys. Chem. Chem. Phys..

[44] Li X., Dai J., Ni D., He X., Zhang H., Zhang J., Fu Q., Liu Y., Lu S. (2020). Insight into the mechanism of allosteric activation of PI3Kα by oncoprotein K-Ras4B. Int. J. Biol. Macromol..

[45] Li X., Ye M., Wang Y., Qiu M., Fu T., Zhang J., Zhou B., Lu S. (2020). How Parkinson’s disease-related mutations disrupt the dimerization of WD40 domain in LRRK2: A comparative molecular dynamics simulation study. Phys. Chem. Chem. Phys..

